# Optimization of a Wedge Shaped T–Type Magnetic Flux Concentrator for High-Sensitivity TMR Sensors

**DOI:** 10.3390/mi17060644

**Published:** 2026-05-23

**Authors:** Guoshuo Peng, Zhenhu Jin, Jiamin Chen

**Affiliations:** 1 State Key Laboratory of Transducer Technology, Aerospace Information Research Institute, Chinese Academy of Sciences, Beijing 100190, China; pengguoshuo20@mails.ucas.ac.cn; 2 School of Electronic, Electrical and Communication Engineering, University of Chinese Academy of Sciences, Beijing 100049, China; 3 College of Materials Sciences and Opto-Electronic Technology, University of Chinese Academy of Sciences, Beijing 100049, China

**Keywords:** tunnel magnetoresistance sensor, magnetic flux concentrator (MFC), magnetic sensor, wedge-shaped structure

## Abstract

A Wedge Shaped T–Type magnetic flux concentrator (MFC) is proposed to improve the magnetic detection capability of tunneling magnetoresistance (TMR) sensors. The TMR chip used in this work integrates a CoFeSiB soft magnetic thin film on-chip and exhibits a sensitivity of 251 mV/Oe with a magnetic noise of 65.3 pT/sqrt(Hz). Based on magnetic circuit analysis and finite-element simulations, the key structural parameters of the Wedge Shaped T–Type MFC were optimized, including the air-gap distance, aspect ratio, and input–output cross-sectional ratio. The optimal parameters were determined as an air gap of 200 μm, an aspect ratio of 2, and a cross-sectional compression ratio exceeding 100. Sixteen MFC structures with different sizes were fabricated and integrated with the TMR sensors for experimental evaluation. The results show that the external flux concentrator does not introduce additional voltage noise while significantly improving the sensor response. With optimized structures, the sensor sensitivity increases from 251 mV/Oe to 17,812 mV/Oe, and the magnetic noise is reduced from 65.3 pT/sqrt(Hz) to 0.92 pT/sqrt(Hz) at 1 Hz. The experimental results demonstrate that the Wedge Shaped T–Type MFC effectively enhances the magnetic field gain and significantly improves the detection limit of TMR sensors.

## 1. Introduction

Tunnel magnetoresistance (TMR) sensors have become important candidates for weak magnetic field detection because of their high sensitivity, room-temperature operation, small size, and low power consumption [1]. With the establishment of coherent tunneling through MgO barriers, the magnetoresistance ratio and sensitivity of TMR devices have been greatly improved, enabling their applications in current sensing, position measurement, and magnetic imaging [2,3]. Compared with SQUID- and OPM-based systems, TMR sensors do not require cryogenic environments or complex optical components. Therefore, they are more suitable for compact and low-cost integration [4,5]. In recent years, TMR sensors have also been explored for ultra-weak biomagnetic measurements such as magnetocardiography and magnetoencephalography [6,7]. However, for pT-level low-frequency magnetic fields, the detection limit of TMR sensors is still constrained by both 1/f noise and insufficient effective magnetic flux coupling [8,9]. Further reduction in the detection limit is difficult to achieve only through film-stack engineering [10], bias optimization, or readout circuit improvement [11].

To improve the effective response of TMR sensors in weak magnetic field detection, magnetic flux concentrators (MFCs) have been introduced into sensor structures [12]. An MFC uses a high-permeability soft magnetic material to collect external magnetic flux and compress it into the sensing gap, thereby enhancing the local magnetic field around the sensor [13,14]. Since this process mainly takes place in the magnetic circuit, it usually does not significantly increase the intrinsic electrical noise of the sensor, and thus can effectively lower the equivalent magnetic noise and improve the detection limit [15]. Previous studies have proposed various MFC configurations, including double-gap, three-dimensional, double-layer tapered, and curved trapezoidal structures, and their effectiveness in improving magnetic gain has been demonstrated [16]. At the same time, it has also been shown that the amplification performance of an MFC strongly depends on the air-gap length, input–output cross-sectional ratio, and overall aspect ratio [17]. Recent parameter studies further suggest that the performance of MFCs is determined more by geometric ratios than by absolute dimensions, which provides clear guidance for structural optimization [18,19].

Although several types of magnetic flux concentrators have been reported, further optimization is still needed for high-sensitivity TMR sensors [20,21]. Conventional rectangular structures are easy to fabricate, but they tend to suffer from reluctance discontinuity, edge leakage, and local demagnetization at abrupt cross-sectional transitions [14]. Increasing the overall size of the concentrator can improve gain, but it also enlarges the planar footprint, increases sensitivity to assembly errors, and makes miniaturization more difficult [19]. Therefore, a new concentrator structure is required to balance high gain, low leakage, and compact integration.

In this work, a Wedge Shaped T-Type MFC is investigated for two main reasons. First, the T-Type structure provides a relatively large input cross-sectional area within a limited planar region, allowing two symmetric magnetic paths to work in parallel, which reduces the equivalent reluctance and improves the efficiency of external flux collection [20]. This geometry is also more compatible with bridge-type TMR chips and is beneficial for balancing gain and device size [19]. Second, the wedge-shaped section enables gradual magnetic flux compression through a continuously varying cross-sectional area, avoiding the abrupt reluctance changes associated with step-like contraction. Compared with simple rectangular or right-angle contraction structures, the wedge transition is more effective in suppressing edge leakage, alleviating local flux crowding, and improving the field uniformity in the sensing gap [17]. Therefore, systematic investigation of the Wedge Shaped T–Type MFC through magnetic circuit analysis, parameter optimization, and experimental verification is expected to provide an effective route for reducing the detection limit of TMR sensors and supporting their applications in weak-field measurements such as magnetocardiography [22].

## 2. System Design and Architecture

In this work, a multi-stage magnetic flux enhancement scheme combining on-chip and off-chip structures is adopted to achieve efficient collection and amplification of weak external magnetic fields. Specifically, an on-chip soft magnetic thin film is employed for primary flux concentration, while an external magnetic flux concentrator further compresses and amplifies the magnetic field, thereby significantly improving the overall magnetic response of the sensor. Building upon this approach, a novel Wedge Shaped T-Type magnetic flux concentrator is proposed. By introducing a wedge-shaped transition into the conventional T-Type structure, the design enables continuous flux compression and efficient transmission in three-dimensional space, effectively mitigating issues such as reluctance discontinuity and flux leakage associated with abrupt geometric transitions. Moreover, this structure fully exploits the three-dimensional space around the sensor, extending the magnetic flux collection path without increasing the planar footprint, thus achieving higher magnetic field gain while maintaining compact device integration.

### 2.1. Performance of the TMR Sensor

**Figure 1:**
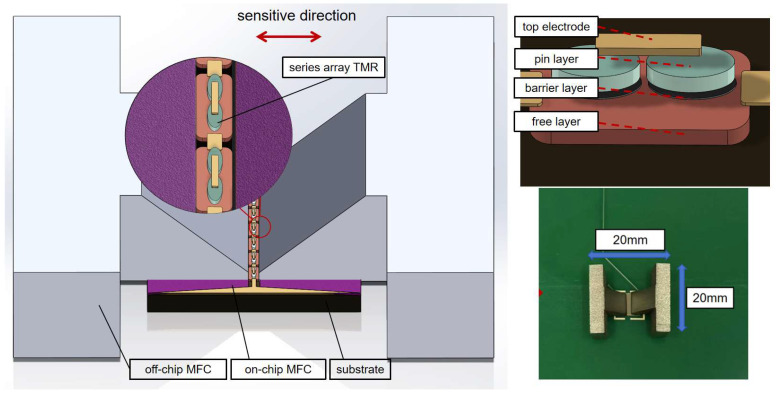
Schematic diagram of an off-chip Wedge Shaped T–Type magnetic flux concentrator for TMR sensor.

The on-chip flux-concentrating thin film was deposited after completing the micro-fabrication of the TMR sensor, and then patterned via a lift-off process. As shown in Figure 1, CoFeSiB soft magnetic thin films are integrated on the TMR grains. In this study, the films were deposited using a magnetron sputtering system, while a pair of magnets was placed on the substrate holder to simultaneously induce magnetic anisotropy in the film. Compared with conventional high-temperature annealing methods for inducing magnetic anisotropy, this magnetically assisted sputtering approach avoids material diffusion in the TMR layers during annealing, thereby ensuring that the intrinsic performance of the TMR is maintained. This on-chip structure enhances flux concentration in the sensing gap, significantly improving sensor sensitivity while maintaining low noise.

**Figure 2:**
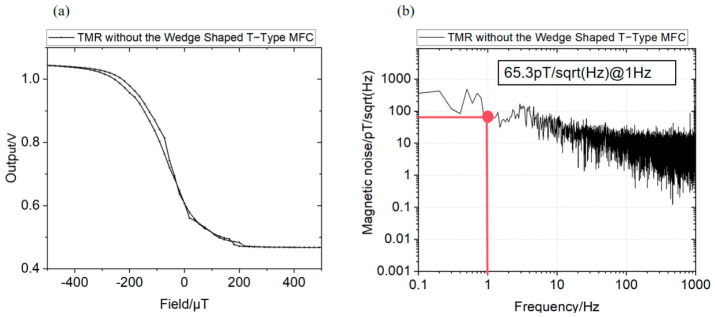
Performance of the bare TMR sensor: (**a**) sensitivity curve; (**b**) magnetic noise spectrum.

Specifically, the bare TMR exhibits a sensitivity of 251 mV/Oe and a magnetic noise level of 65.3 pT/sqrt(Hz). As shown in Figure 2, both the output response curve and the magnetic noise spectrum demonstrate the sensor’s high capability for detecting weak magnetic fields. These properties provide a reliable basis for the subsequent integration and evaluation of external wedge-shaped flux concentrators.

### 2.2. Design of the Wedge Shaped T–Type MFC

To further improve the performance of the tunneling magnetoresistance (TMR) sensor, a Wedge Shaped T–Type MFC is proposed in this study. This structure exhibits improved magnetic flux concentration capability. The schematic diagram of the TMR sensor integrated with the Wedge Shaped T–Type MFC is shown in Figure 1.

By incorporating the wedge-shaped design, magnetic flux can be more effectively concentrated within a specific region, thereby reducing magnetic flux leakage and improving the overall utilization efficiency of the magnetic field. In addition, the wedge geometry helps optimize the magnetic field distribution within the air gap, enhancing field uniformity and consequently improving device performance. Furthermore, due to the more efficient magnetic flux concentration, the wedge-shaped structure can reduce eddy current losses and other forms of energy dissipation, which is expected to improve magnetic flux utilization. Meanwhile, the wedge design allows a reduction in material usage while maintaining effective flux concentration, which is beneficial for compact integration.

## 3. Magnetic Circuit Theoretical Analysis

When establishing the equivalent magnetic circuit model, the magnetic flux transmission path of the Wedge Shaped T–Type MFC can be represented by a combination of magnetic reluctances connected in series and parallel. Due to the geometric symmetry of the structure, the magnetic flux collected by the two input blocks is guided through the wedge-shaped transition arms toward the central junction region and finally concentrated into the sensing air gap. Consequently, the two symmetric flux paths can be regarded as parallel branches, while each branch consists of three main sections: the input rectangular block, the wedge-shaped transition region, and the output region close to the central junction.

**Figure 3:**
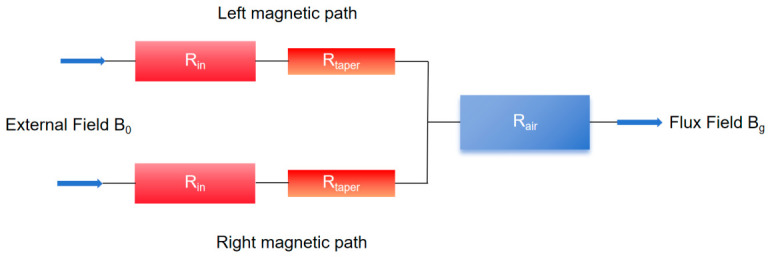
Schematic Diagram of The Equivalent Magnetic Circuit Model.

The proposed flux concentrator consists of two symmetric magnetic flux paths. Each path is composed of three sections connected in series: an input rectangular soft magnetic block (the wide tail section of the T-shaped structure), a wedge-shaped transition section where the cross-sectional area gradually changes from the input to the output, and a central air gap where the TMR sensor is located nearby. Due to the structural symmetry, the two magnetic paths operate in parallel from the perspective of the magnetic circuit, jointly supplying magnetic flux to the central air gap. Figure 3 shows the equivalent magnetic circuit of the Wedge Shaped T–Type MFC. Although the diagram is not a physically closed loop, it represents the continuous magnetic flux path: the input block, wedge-shaped transition, and air gap form each series magnetic path, and the two symmetric paths operate in parallel, jointly supplying flux to the central sensing gap. This equivalent magnetic circuit can be used to formulate magnetic circuit equations for gain calculation.

### 3.1. Magnetic Circuit Model and Parameter Definition

The proposed the Wedge Shaped T–Type MFC consists of two symmetric flux paths. Each path comprises three sections connected in series: an input rectangular soft-magnetic block (wide tail of the T-shaped structure) with a constant cross-section, a wedge-shaped transition section where the cross-sectional area gradually changes from the input to the output, and a central air gap where the TMR sensor is located nearby. Owing to the symmetry, the two paths operate in parallel in terms of the magnetic circuit and jointly deliver magnetic flux to the air gap.

Due to the structural symmetry, the two magnetic paths operate in parallel in terms of the magnetic circuit and jointly supply magnetic flux to the central air gap. The input and output cross-sectional areas are defined as:

(1) $$D_{i}=w_{i}t_{i}$$

where $w_{i}$ and $t_{i}$ denote the width and thickness of the input section.

(2) $$D_{o}=w_{o}t_{o}$$

where $w_{o}$ and $t_{o}$ represent the width and thickness of the wedge end.

The geometric compression ratio is defined as:

(3) $$\eta =\frac{D_{o}}{D_{i}},(0<\eta \leq 1)$$

Although the wedge structure mainly varies in thickness in the practical design, the theoretical analysis only requires the cross-sectional area to change from $D_{i}$ to $D_{o}$. Therefore, no additional assumptions about constant width or thickness are required.

### 3.2. Magnetic Circuit Relation

According to magnetic circuit theory:

(4) $$\Phi =\frac{F}{R_{tot}}$$

where Φ is the magnetic flux, F is the magnetomotive force (MMF), $R_{tot}$ is the total magnetic reluctance.

Equation (5) represents the reluctance of a single magnetic path, composed of the input rectangular soft-magnetic block and the wedge-shaped transition segment:

(5) $$R_{path}={R_{in}+R}_{taper}$$

Equation (6) expresses the equivalent reluctance of the two symmetric paths in parallel. Since the two branches are identical, their parallel combination leads to:

(6) $$R_{LR}=\frac{{R_{in}+R}_{taper}}{2}$$

Since the air gap reluctance is in series with $R_{LR}$, the total reluctance is:

(7) $$R_{tot}=R_{LR}+R_{g}=\frac{R_{in}+R_{taper}}{2}+R_{g}$$

The reluctance of the input rectangular block is:

(8) $$R_{in}=\frac{l_{in}}{\mu_{0}\mu_{r}D_{i}}$$

For the wedge transition of length L, assuming a linear variation in cross-sectional area,

(9) $$A(x)=D_{i}-(D_{i}-D_{o})\frac{x}{L}$$

the reluctance is:

(10) $$R_{taper}=\int_{0}^{L}\frac{dx}{\mu_{0}\mu_{r}A(x)}=\frac{L}{\mu_{0}\mu_{r}(D_{i}-D_{o})}ln(\frac{D_{i}}{D_{o}})$$

Using $\eta =D_{o}/D_{i}$, it becomes:

(11) $$R_{taper}(\eta )=\int_{0}^{L}\frac{dx}{\mu_{0}\mu_{r}A(x)}=\frac{L}{\mu_{0}\mu_{r}(D_{i}\left(1-\eta \right))}ln(\frac{1}{\eta })$$

The air-gap reluctance is:

(12) $$R_{g}=\int_{0}^{L}\frac{dx}{\mu_{0}A_{g}}$$

where g is the gap length and $A_{g}$ is the effective gap area (a fringing correction can be included via $A_{g}^{eff}=k_{f}A_{g}$ if needed).

The magnetic flux density in the gap is:

(13) $$B_{g}=\frac{\Phi }{A_{g}}$$

For an external field $B_{0}$, $H_{0}=B_{0}/\mu_{0}$, and the MMF can be expressed as:

(14) $$F=H_{0}l_{eff}=\frac{B_{0}}{\mu_{0}}l_{eff}$$

where $l_{eff}$ is the effective length along the equivalent magnetic path. Substitution yields:

(15) $$B_{g}=\frac{B_{0}l_{eff}}{\mu_{0}A_{g}R_{tot}}$$

Defining the flux concentration gain as:

(16) $$G=\frac{B_{g}}{B_{0}}$$

(17) $$G\left(\eta \right)=\frac{l_{eff}}{\mu_{0}A_{g}\left[\frac{R_{in}+R_{taper(\eta )}}{2}+R_{g}\right]}$$

For the Wedge Shaped T–Type flux concentrator, the relationship between the input-to-output cross-sectional area ratio and magnetic field gain is not linear, but instead exhibits a clear nonlinear behavior. This nonlinearity mainly arises from the variation in magnetic reluctance in the tapered transition region, leading to a non-uniform enhancement of flux gain with respect to the geometric compression ratio.

## 4. Simulation Design

**Figure 4:**
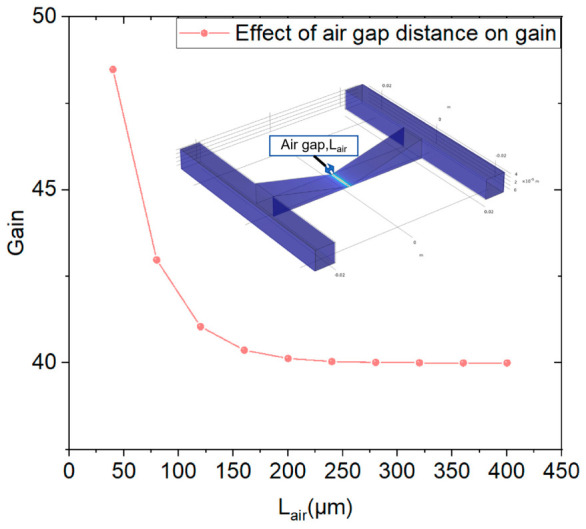
Effect of Air Gap Distance on the Gain of the Wedge Shaped T–Type MFC.

To systematically investigate the performance of the Wedge Shaped T–Type MFC, we first conducted detailed simulations to evaluate the effect of the air gap distance on the flux concentration gain, as shown in Figure 4. The detailed simulation parameters are listed in Table 1. In the simulation model, all geometric parameters and material properties of the flux concentrator were kept constant, while the air gap distance $L_{air}$ was varied stepwise to compute the corresponding magnetic flux gain in the air gap. The gain decreases rapidly when the air gap is below 200 μm and becomes nearly stable when the gap exceeds 200 μm. When the air gap is small (less than 200 μm), the gain decreases rapidly with increasing gap distance. This is primarily because the air gap reluctance occupies a significant portion of the total magnetic circuit reluctance; as the air gap increases, the total reluctance increases, reducing the magnetic flux density in the sensing region. However, when the air gap exceeds 200 μm, the gain variation becomes minor and the curve approaches a plateau, indicating that further increases in the air gap have limited effect on flux compression and concentration, and the magnetic circuit operates in a stable regime.

**Figure 5:**
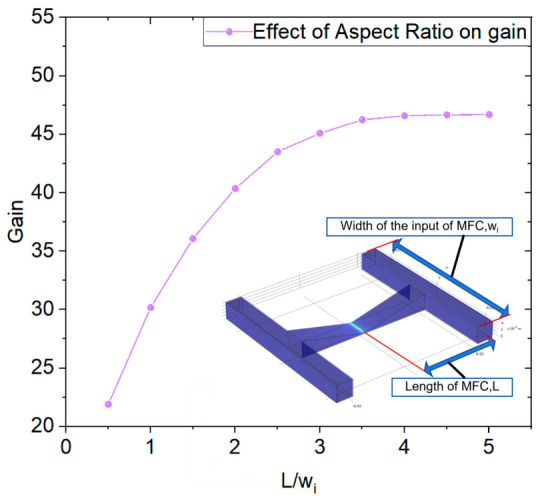
Effect of Aspect Ratio on the Gain of the Wedge Shaped T–Type MFC.

**Figure 6:**
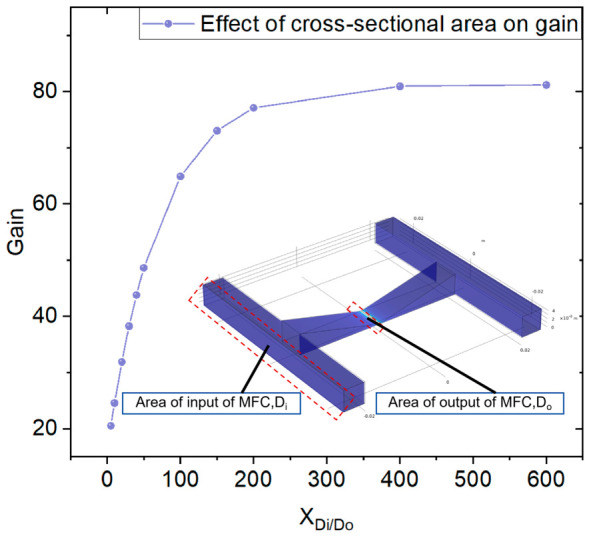
Effect of the Input–Output Cross-Sectional Area Ratio on the Gain of the Wedge Shaped T–Type MFC.

Based on this observation, as shown in Figure 4, to facilitate experimental comparison and minimize the influence of assembly errors, the air gap distance of the flux concentrator was fixed at 200 μm in all subsequent simulations and experiments. For Figure 4, Figure 5 and Figure 6, the vertical axis labeled “Gain” represents a linear ratio. This setting ensures that the gain approaches the theoretical stable value while maintaining repeatability and comparability of experimental data. Furthermore, these simulation results provide guidance for optimizing other structural parameters, such as the ratio between the output and input cross-sectional areas ($D_{i}/D_{o}$) and the wedge section length or angle, all of which can be adjusted while maintaining a reasonable air gap. Overall, this simulation study clarifies the effect of the air gap distance on the flux concentration gain and provides a solid theoretical basis for subsequent experimental design.

To investigate the influence of geometric parameters on the performance of the Wedge Shaped T–Type MFC, simulations were conducted to analyze the relationship between the aspect ratio $L/w_{i}$ and the flux concentration gain, as shown in Figure 5. In the analysis shown in Figure 5, where the aspect ratio is varied, the air-gap width is kept constant. The results indicate that when the aspect ratio is smaller than 2, the gain increases rapidly with increasing aspect ratio. This is because a longer flux concentrator can collect and guide more magnetic flux toward the sensing air gap, resulting in a higher magnetic flux density in the gap region. However, when the aspect ratio exceeds 2, the growth rate of the gain becomes much slower and gradually approaches saturation, indicating that further increasing the length of the flux concentrator provides only limited improvement in flux concentration performance. Considering the trade-off between gain performance and the overall size of the device, an aspect ratio of 2 was selected as the optimal design parameter. This configuration ensures a relatively high flux concentration gain while reducing the planar size of the concentrator, which is beneficial for the miniaturization of the TMR sensor.

To investigate the influence of the cross-sectional area ratio on the flux concentration performance of the Wedge Shaped T–Type MFC, simulations were conducted to analyze the relationship between the input–output cross-sectional area ratio $D_{i}/D_{o}$ and the gain, as shown in Figure 6. The simulation results indicate that the gain increases with the increase in the cross-sectional area ratio. This behavior can be attributed to the wedge-shaped structure, which utilizes the longitudinal space to gradually compress the magnetic flux, thereby guiding more magnetic flux toward the sensing air gap and enhancing the magnetic field intensity in this region.

When the cross-sectional ratio $D_{i}/D_{o}$ is smaller than 100, the gain increases rapidly with increasing ratio. In this range, the input cross-sectional area is significantly larger than the output area, resulting in a strong flux compression effect. Consequently, a large amount of magnetic flux is concentrated in the sensing region, leading to a substantial increase in magnetic field gain. When the ratio lies between 100 and 200, the growth rate of the gain gradually decreases. Although the flux compression effect continues to increase with the cross-sectional ratio, the magnetic reluctance distribution of the magnetic circuit begins to approach a stable state, and the improvement in flux concentration efficiency becomes less significant. When the cross-sectional ratio exceeds 200, the gain curve gradually approaches saturation and exhibits a clear marginal effect. This phenomenon occurs because the wedge structure has already provided sufficient flux compression, and the magnetic field in the air gap approaches the maximum level that the magnetic circuit can supply. Meanwhile, the magnetic permeability of the soft magnetic material and the flux transmission capability limit further improvement in flux concentration, making additional increases in the cross-sectional ratio ineffective.

Based on the above simulation analysis, the influence of key structural parameters of the Wedge Shaped T–Type MFC on the flux concentration gain was systematically investigated. The results indicate that when the air gap distance exceeds 200 μm, the gain variation becomes small and tends to stabilize. Therefore, the air gap distance was fixed at 200 μm in the subsequent study to minimize the influence of assembly errors on the experimental results. In addition, the gain increases rapidly when the aspect ratio is smaller than 2, while the growth rate gradually decreases when the aspect ratio exceeds 2. Considering the trade-off between magnetic gain and device size, an aspect ratio of 2 was selected as the optimized design parameter. Furthermore, increasing the cross-sectional area ratio $D_{i}/D_{o}$ significantly enhances the flux concentration performance, but the gain gradually approaches saturation when the ratio exceeds 200. Based on these simulation results, the key structural parameters of the Wedge Shaped T–Type MFC were determined. Subsequently, device fabrication and experimental measurements were carried out to further verify the flux concentration performance of the proposed structure.

## 5. Results and Discussion

The previous simulation analysis identified the key structural parameters of the Wedge Shaped T–Type MFC and provided an optimized design for enhancing flux concentration performance. The simulation results indicate that appropriate design of the air gap distance, the aspect ratio of the concentrator, and the input–output cross-sectional area ratio can significantly increase the magnetic field intensity in the sensing gap, thereby improving the sensitivity of magnetoresistance sensors. However, experimental verification is required to evaluate the feasibility and practical performance of the proposed structure. Therefore, the optimized Wedge Shaped T–Type flux concentrator was fabricated and integrated with the TMR sensor for experimental evaluation. An experimental measurement platform is established to compare the sensor output signals before and after integrating the flux concentrator. Through this comparison, the magnetic field enhancement capability of the proposed structure is validated, and its potential for weak magnetic field detection applications is evaluated.

During device fabrication and assembly, careful measures were taken to ensure the structural accuracy of the magnetic flux concentrator and the reliability of experimental results. The Wedge Shaped T–Type magnetic flux concentrators were fabricated using laser cutting, which provides high dimensional consistency and repeatability. The overall fabrication tolerance was controlled within 1 mm, meeting the design requirements. For assembly, all devices were aligned under a microscope to ensure precise positioning between the TMR sensor and the flux concentrator. The air-gap distance was carefully controlled to be within 200 μm, while maintaining structural symmetry as much as possible. Considering practical limitations in manual assembly, a tolerance of approximately ±50 μm in the air-gap distance was present. According to the simulation results presented earlier, when the air-gap distance is around 200 μm, the magnetic flux gain reaches a relatively stable regime, and small variations in the gap have only a minor effect on the gain. Therefore, the ±50 μm assembly tolerance does not significantly influence the flux concentration performance. This effectively reduces the impact of assembly induced variations on the experimental results and ensures good reliability and repeatability of the measurements. To ensure that the TMR chip and the magnetic flux concentrator are positioned on the same horizontal plane, a silicon spacer with a thickness equal to that of the TMR wafer was introduced beneath the flux concentrator. This configuration effectively eliminates height mismatch and prevents distortion of the magnetic field distribution caused by vertical misalignment, thereby improving assembly consistency. In addition, a non-magnetic adhesive tape was used to fix the flux concentrator in place, avoiding any magnetic interference while ensuring mechanical stability during measurements.

**Figure 7:**
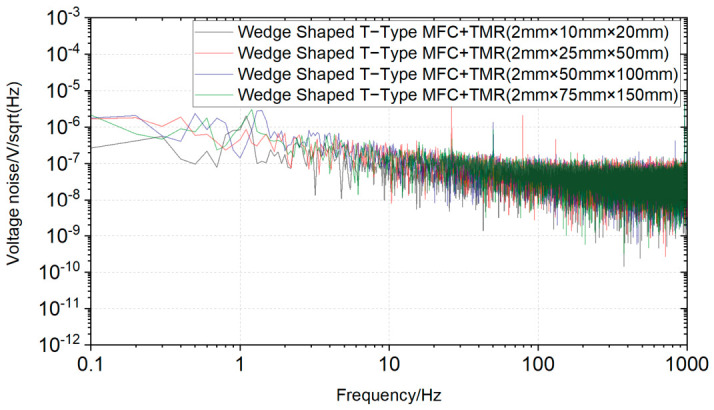
Voltage Noise Spectra of TMR Sensors Integrated with Wedge Shaped T–Type MFC of Different Sizes.

In this work, sixteen T-shaped magnetic flux concentrators with different dimensions were designed and fabricated. The detailed dimensions are listed in Table 2. Each concentrator was integrated with a high-sensitivity TMR chip that already includes an on-chip flux concentrating thin film. As shown in Figure 7, integrating flux concentrators of different dimensions does not introduce additional voltage noise over the measured frequency range, and the noise spectra remain nearly identical for all configurations. This is primarily because the flux concentrators are passive soft magnetic structures that do not participate in electrical signal conduction and do not generate resistive or thermal noise, thus leaving the intrinsic noise characteristics of the TMR sensor unaffected.

**Figure 8:**
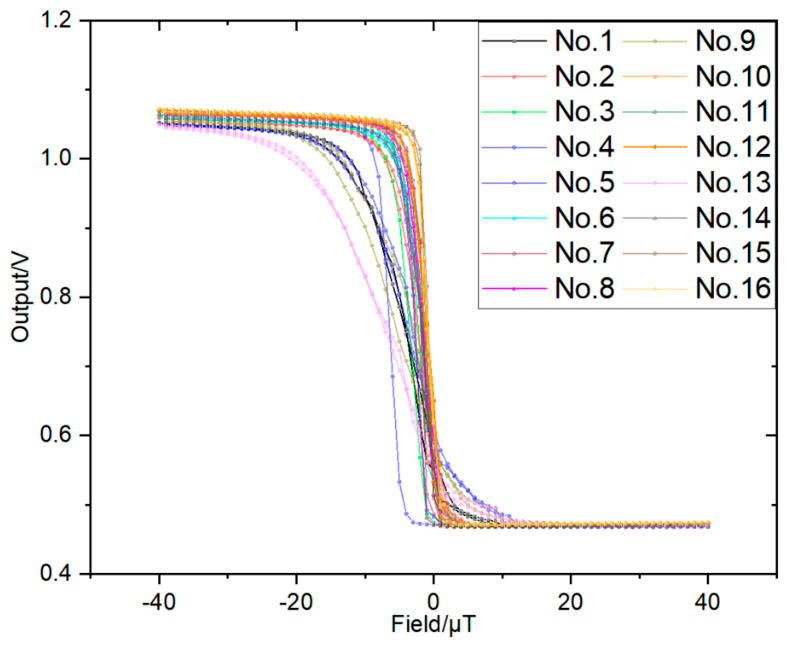
Magnetic Response of TMR Sensors Integrated with Wedge Shaped T–Type MFC of Different Sizes.

**Figure 9:**
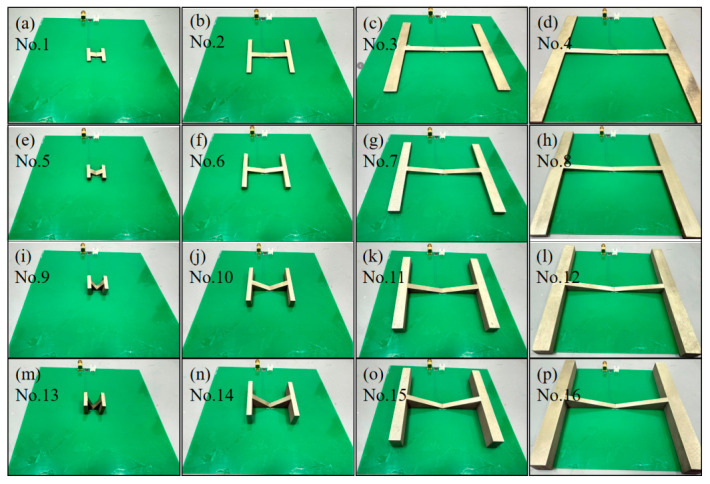
TMR Sensors Integrated with the 16 sizes of the Wedge Shaped T–Type MFC.

Consequently, with the noise level remaining unchanged, the magnetic field gain provided by the flux concentrator can directly enhance the sensor’s magnetic field detection capability, as shown in Figure 8. In other words, the ultimate magnetic detection limit of the TMR sensor is positively correlated with the gain of the flux concentrator, confirming the feasibility of improving weak magnetic field detection performance through structural optimization while maintaining low-noise operation. The physical photographs of wedge-shaped T-type MFC+TMR with different dimensions are shown in Figure 9.

**Figure 10:**
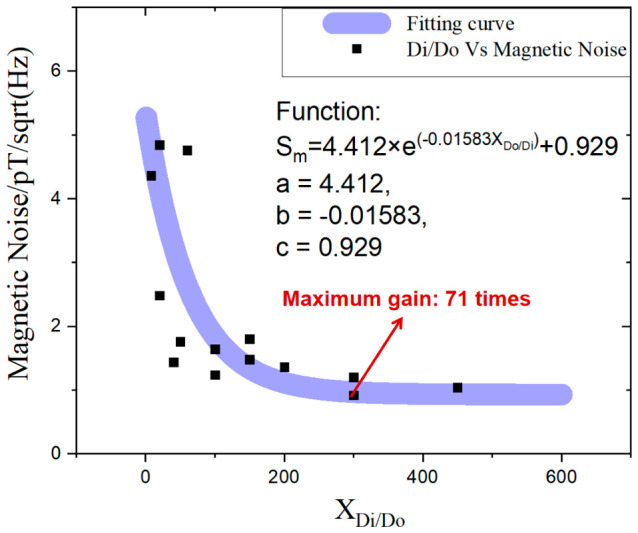
The Influence of the Input–output Cross-sectional Area Ratio on the Magnetic Noise of the TMR Sensor.

This study investigates the influence of the input–output cross-sectional area ratio on the magnetic detection limit of the TMR sensor, as shown in Figure 10. The experimental results indicate that the magnetic noise of the TMR device decreases exponentially with increasing cross-sectional ratio $D_{i}/D_{o}$. When the ratio is relatively small, the magnetic noise decreases rapidly, indicating that the flux concentrator effectively enhances the magnetic field intensity in the sensing gap and significantly improves the magnetic detection capability of the sensor. As the ratio increases to approximately 100, a noticeable inflection point appears in the noise curve, suggesting that the flux compression capability of the concentrator approaches its effective operating limit.

With further increase in the cross-sectional ratio, the reduction rate of magnetic noise gradually slows down. When the ratio exceeds 200, the noise level tends to stabilize, exhibiting a clear marginal effect. This phenomenon indicates that although increasing the cross-sectional ratio initially leads to significant improvement in noise performance, further enlargement of the ratio becomes less effective due to the stabilization of the magnetic reluctance distribution in the magnetic circuit and the limitations imposed by the permeability of the soft magnetic material.

Based on the experimental data, an exponential fitting was performed to describe the relationship between the cross-sectional ratio and the magnetic noise of the Wedge Shaped T–Type MFC. The empirical expression is given as:

(18) $$S_{B}=4.412e^{-0.01583X_{\frac{Di}{Do}}}+0.929$$

where $S_{B}$ represents the magnetic noise level of the TMR device. This empirical model accurately describes the variation trend of magnetic noise with respect to the cross-sectional ratio and provides a useful guideline for optimizing the flux concentrator design.

**Figure 11:**
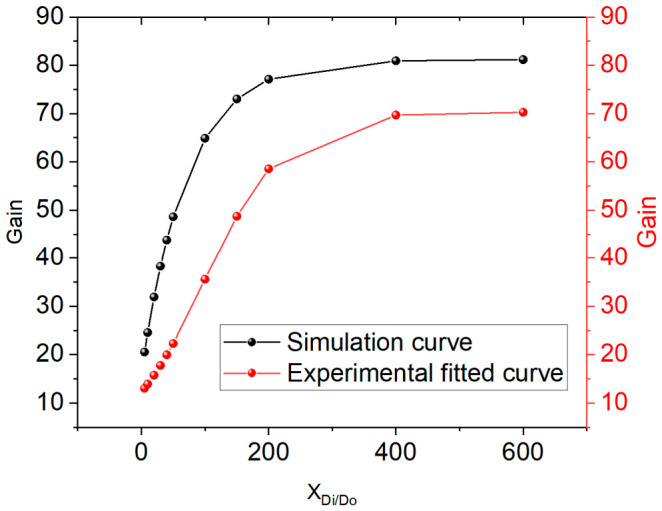
The Influence of the Input–output Cross-sectional Area Ratio on the Gain of the TMR Sensor.

As shown in Figure 11, the gain variation in the flux concentrator under different input/output cross-section ratios is presented, including both simulation curves and experimentally fitted curves. The overall trend of gain variation for the actual Wedge Shaped T–Type flux concentrator is consistent with the simulation results; however, the measured gain is slightly lower than the simulated predictions. This discrepancy may be attributed to minor fabrication imperfections, variations in material properties, and edge leakage effects in the actual device, which are not fully captured in the simulations.

The combined simulation and experimental results indicate that the input-to-output cross-sectional ratio of the Wedge Shaped T–Type magnetic flux concentrator plays a dominant role in determining device detectivity. As this ratio increases, the wedge structure becomes more effective in compressing and guiding magnetic flux, allowing a larger portion of the external magnetic field to be concentrated into the air-gap region. This leads to a higher local magnetic field and a lower equivalent magnetic noise, thereby improving the overall detectivity of the sensor. The results show that the best detectivity is typically obtained when the cross-sectional ratio is around 200. Beyond this region, the improvement becomes much less pronounced and gradually approaches saturation. This suggests that increasing the geometric compression ratio is highly effective in the low-to-intermediate range, whereas the magnetic field gain no longer increases proportionally once the ratio becomes sufficiently large.

This behavior is closely related to both magnetic circuit distribution and the intrinsic properties of the soft magnetic material. As the cross-sectional ratio increases, the reluctance of the air gap gradually becomes the dominant component in the overall magnetic circuit, making the air gap the main bottleneck for further flux enhancement. Under this condition, a further increase in geometric compression cannot significantly raise the magnetic flux density in the sensing region. At the same time, the soft magnetic material tends to approach magnetic saturation at high flux density, which reduces its effective permeability and limits further gain improvement. Excessively large cross-sectional ratios may also introduce stronger flux crowding and edge leakage, lowering the overall efficiency of flux utilization. Taking into account flux compression capability, magnetic circuit matching, material utilization, and practical fabrication and assembly considerations, the input-to-output cross-sectional ratio of the Wedge Shaped T–Type magnetic flux concentrator is best designed within the range of 100 to 300. Within this window, a good balance can be achieved between performance, robustness, and engineering feasibility, while a ratio around 200 usually provides a near-optimal trade-off between detectivity and structural stability.

To more comprehensively characterize the performance of TMR devices integrated with a two-stage magnetic flux concentrator (MFC), an alternating magnetic field response experiment was conducted. In the experiment, a Helmholtz coil was used to generate an alternating magnetic field with an amplitude of 1 nT over a frequency range of 1–10,000 Hz. A data acquisition card was employed to measure the output response of TMR sensors integrated with T-shaped wedge MFCs of different sizes.

**Figure 12:**
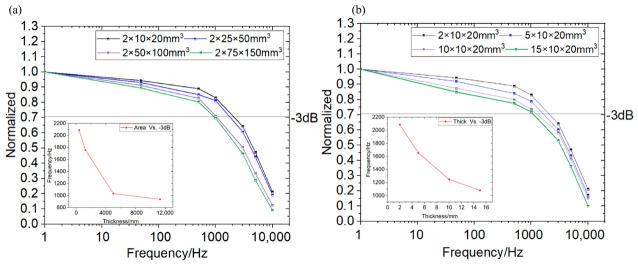
(**a**) Frequency response results of TMR sensors integrated with MFCs of different planar sizes. (**b**) Frequency response results of TMR sensors integrated with MFCs of different thicknesses.

As shown in Figure 12a, under the condition of constant thickness and aspect ratio, the gain of the MFC exhibits more pronounced frequency-dependent attenuation as the planar size increases. When the planar size is 2000 mm^2^ (10 mm × 20 mm), the −3 dB cutoff frequency is 2087 Hz. When the planar size increases to 11,250 mm^2^ (75 mm × 150 mm), the −3 dB frequency decreases to 938 Hz. This phenomenon arises from the combined effects of eddy currents and the skin effect. With increasing planar dimensions, larger-scale eddy current loops are more easily formed within the material, producing stronger opposing magnetic fields that shield the applied external field. Meanwhile, at higher frequencies, the magnetic field becomes increasingly confined to the surface region of the material, making it difficult for the interior to effectively participate in magnetic flux conduction, thereby reducing high-frequency magnetic flux transmission capability. As shown in Figure 12b, when the planar size is kept constant (2000 mm^2^), increasing the thickness of the wedge-shaped MFC also leads to a faster attenuation of high-frequency gain. When the thickness is 2 mm, the −3 dB frequency is 2087 Hz; when the thickness increases to 15 mm, the −3 dB frequency decreases to 1079 Hz. This phenomenon is primarily dominated by the skin effect. As frequency increases, the effective penetration depth of the magnetic field in conductive soft magnetic materials decreases. When the structure thickness increases, the effective region participating in magnetic conduction becomes gradually confined to the surface layer, leading to a reduction in equivalent magnetic permeability. For the Wedge Shaped T–Type MFC, this effect accumulates along the wedge transition path, further weakening the magnetic flux concentration capability at high frequencies.

## 6. Conclusions

This work presents a Wedge Shaped T–Type MFC for improving the magnetic detection capability of TMR sensors. Magnetic circuit analysis and simulations were carried out to investigate the influence of key structural parameters of the concentrator, including the air-gap distance, aspect ratio, and input–output cross-sectional ratio. The results show that the flux concentration gain becomes stable when the air gap exceeds 200 μm. The gain increases rapidly when the aspect ratio is smaller than 2, while the improvement gradually slows down as the aspect ratio further increases. The cross-sectional ratio also plays an important role in flux concentration. When the ratio is below 100, the gain increases rapidly, while it gradually saturates when the ratio exceeds 200.

Based on these findings, 16 Wedge Shaped T–Type MFCs with different dimensions were fabricated and integrated with a high-sensitivity TMR chip that includes an on-chip CoFeSiB soft magnetic thin film. Experimental results show that the external concentrator does not introduce additional voltage noise and significantly enhances the sensor response. With the optimized structure, the sensitivity increases from 251 mV/Oe to 17,812 mV/Oe, while the magnetic noise at 1 Hz decreases from 65.3 pT/sqrt(Hz) to 0.92 pT/sqrt(Hz), which achieves a maximum gain of 71 times. These results demonstrate that optimizing the wedge-shaped flux concentrator structure is an effective way to improve the detection limit of TMR sensors for weak magnetic field measurements.

Compared with representative published studies, the detectivity of 0.92 pT/sqrt(Hz) at 1 Hz achieved in this work places the device among the leading room-temperature TMR sensors for low-frequency weak-field detection, and at the same order of magnitude as the representative sub-pT results reported so far. A quantitative performance comparison of the developed sensor against existing advanced TMR devices for weak magnetic field sensing is illustrated in Table 3. A cross-comparison further suggests that sub-pT performance is typically achieved with the assistance of magnetic flux concentration, while device size strongly affects flux collection capability, gain ceiling, and application flexibility. In contrast to previously reported planar concentrator schemes with relatively fixed geometries, the Wedge Shaped T–Type magnetic flux concentrator proposed here offers a larger structural design space and more effective use of three-dimensional flux compression. This allows the input-to-output cross-sectional ratio to be optimized within the range of 100 to 300, providing a good balance among detectivity, structural size, and engineering feasibility. Meanwhile, the −3 dB bandwidth of the TMR integrated with the Wedge Shaped T–Type MFC can reach the kilohertz range, which is sufficient to meet the requirements for biomedical weak magnetic signal detection. Overall, the achieved 0.92 pT/√Hz not only demonstrates that this work reaches the first tier of room-temperature TMR performance, but also highlights the broader value of the multi-stage flux enhancement strategy combined with wedge-shaped geometric optimization.

## Figures and Tables

**Table 1:** Simulation Parameters of the Wedge Shaped T–Type Flux Concentrator.

Name	Parameter Value	Description
T_i_	2~15 mm	Thickness of input
T_o_	1 mm	Thickness of output
w_i_	20~150 mm	Width of input
w_o_	6.5 mm	Width of output
L	20~150 mm	Length of MFC
Gap	50~400 mm	Length of gap
B_i_	1 mT	Magnetic field of input

**Table 2:** Performance Summary of TMR Sensors Integrated with the Wedge Shaped T–Type MFC.

	MFC Size mm^3^	Sensitivity mV/Oe	D_i_/D_o_	Magnetic Noise pT/sqrt (Hz)@1 Hz
TMR	/	251	/	65.32
No. 1	2 × 10 × 20	4610	8	4.36
No. 2	2 × 25 × 50	7350	20	2.48
No. 3	2 × 50 × 100	12,542	40	1.44
No. 4	2 × 75 × 150	14,375	60	1.04
No. 5	5 × 10 × 20	4673	20	4.84
No. 6	5 × 25 × 50	9640	50	1.76
No. 7	5 × 50 × 100	14,620	100	1.24
No. 8	5 × 75 × 150	12,189	150	1.48
No. 9	10 × 10 × 20	2873	80	6.28
No. 10	10 × 25 × 50	11,526	100	1.64
No. 11	10 × 50 × 100	12,730	200	1.36
No. 12	10 × 75 × 150	13,501	300	1.2
No. 13	15 × 10 × 20	3285	60	4.76
No. 14	15 × 25 × 50	9209	150	1.8
No. 15	15 × 50 × 100	17,812	300	0.92
No. 16	15 × 75 × 150	13,933	450	1.04

**Table 3:** Comparison of this work with representative TMR weak magnetic field sensors.

Work	Year	Approach	Magnetic Noise	Size	Ref.
This work	2026	On-chip CoFeSiB soft magnetic film + off-chip Wedge Shaped T–Type MFC	0.92 pT/sqrt(Hz)	2 × 10 × 20~15 × 75 × 150 mm^3^;	/
Cardoso	2014	permanent magnet bias + flux guides	49 pT/sqrt(Hz)	Active area ~400 × 6 μm^2^	[5]
Nguyen	2018	TMR sensor with AC magnetic field modulation	5~10 pT/sqrt(Hz)	/	[7]
Nakano	2025	Sub-pT TMR design with MFC for biomagnetic detection	0.94 pT/sqrt(Hz)	26 × 26 mm^2^	[23]

## Data Availability

The data presented in this study are available on reasonable request from the corresponding author due to privacy.
